# Two-dimensional correlation spectroscopy in polymer study

**DOI:** 10.3389/fchem.2015.00014

**Published:** 2015-03-11

**Authors:** Yeonju Park, Isao Noda, Young Mee Jung

**Affiliations:** ^1^Department of Chemistry, Kangwon National UniversityChunchon, South Korea; ^2^Department of Materials Science and Engineering, University of DelawareNewark, DE, USA

**Keywords:** two-dimensional correlation spectroscopy, 2DCOS, polymer, hetero-spectral correlation, projection 2D, PCA 2DCOS, eigenvalue manipulating transformation, self-modeling curve resolution

## Abstract

This review outlines the recent works of two-dimensional correlation spectroscopy (2DCOS) in polymer study. 2DCOS is a powerful technique applicable to the in-depth analysis of various spectral data of polymers obtained under some type of perturbation. The powerful utility of 2DCOS combined with various analytical techniques in polymer studies and noteworthy developments of 2DCOS used in this field are also highlighted.

## Introduction

Noda developed two-dimensional correlation spectroscopy (2DCOS) for analyzing the small-amplitude dynamic strain-dependent time-resolved IR linear dichroism spectra of a polymer film (Noda, 1986). Since the concept of 2DCOS was expanded to the various spectroscopic applications in 1993 (Noda, 1993), the generalized 2DCOS has become a very powerful analytical technique in many fields of spectroscopic studies, especially in polymer study.

The generalized 2DCOS can elucidate information in spectral variations, e.g., IR (Kim et al., 2006b; Cerdà-Costa et al., 2009; Huang et al., 2009; Unger et al., 2009, 2011; Del Río et al., 2010; Huang and Kuo, 2010; Jelèić et al., 2010; Jia et al., 2010; Lee et al., 2010, 2012; Peng et al., 2010; Popescu and Vasile, 2010, 2011; Zheng et al., 2010; Cheng et al., 2011; Jin et al., 2011; Kuo and Liu, 2011; Musto et al., 2011; Quaroni et al., 2011; Wang and Wu, 2011; Zhang et al., 2011; Ando et al., 2012; Qu et al., 2012; Su et al., 2012; Wu et al., 2012; Chai et al., 2013; Lai and Wu, 2013; Park et al., 2013; Shinzawa et al., 2013; Wang et al., 2013, 2014; Galizia et al., 2014; Hou et al., 2014; Noda, 2014c; Seo et al., 2014), Raman (Radice et al., 2010; Tang et al., 2010; Ma et al., 2011; Ji et al., 2012; Pazderka and Kopecký Jr, 2012; Brewster et al., 2013; Grzeszczuk et al., 2013; Noda, 2014d), terahertz (THz) (Hoshina et al., 2012, 2014), X-ray (Guo et al., 2011), UV-Vis (Hong et al., 2005; Jiang and Wu, 2008; Sikirzhytski et al., 2012; Zhong et al., 2012), NMR (Oh et al., 2009; Li et al., 2013), fluorescence (Hur et al., 2011; Zhang et al., 2013), and even chromatography (Izawa et al., 2001), under various external perturbations, such as thermal, electrical, optical, magnetic, and chemical perturbations (Noda, 1986, 1993; Hong et al., 2005; Kim et al., 2006b; Jiang and Wu, 2008; Cerdà-Costa et al., 2009; Huang et al., 2009; Oh et al., 2009; Unger et al., 2009; Del Río et al., 2010; Huang and Kuo, 2010; Jelèić et al., 2010; Jia et al., 2010; Lee et al., 2010, 2012; Peng et al., 2010; Popescu and Vasile, 2010, 2011; Radice et al., 2010; Tang et al., 2010; Zheng et al., 2010; Zhang et al., 2011; Cheng et al., 2011; Guo et al., 2011; Jin et al., 2011; Kuo and Liu, 2011; Ma et al., 2011; Quaroni et al., 2011; Unger et al., 2011; Wang and Wu, 2011; Ando et al., 2012; Hoshina et al., 2012, 2014; Ji et al., 2012; Pazderka and Kopecký Jr, 2012; Qu et al., 2012; Sikirzhytski et al., 2012; Su et al., 2012; Wu et al., 2012; Zhong et al., 2012; Brewster et al., 2013; Chai et al., 2013; Grzeszczuk et al., 2013; Lai and Wu, 2013; Li et al., 2013; Park et al., 2013; Shinzawa et al., 2013; Wang et al., 2013, 2014; Hou et al., 2014; Noda, 2014c,d; Seo et al., 2014). IR spectroscopy is the most common analytical probes used in 2DCOS (Kim et al., 2006b; Cerdà-Costa et al., 2009; Huang et al., 2009; Unger et al., 2009, 2011; Del Río et al., 2010; Huang and Kuo, 2010; Jelèić et al., 2010; Jia et al., 2010; Lee et al., 2010, 2012; Peng et al., 2010; Popescu and Vasile, 2010, 2011; Zheng et al., 2010; Cheng et al., 2011; Jin et al., 2011; Kuo and Liu, 2011; Musto et al., 2011; Quaroni et al., 2011; Wang and Wu, 2011; Zhang et al., 2011; Ando et al., 2012; Qu et al., 2012; Su et al., 2012; Wu et al., 2012; Chai et al., 2013; Lai and Wu, 2013; Park et al., 2013; Shinzawa et al., 2013; Wang et al., 2013, 2014; Galizia et al., 2014; Hou et al., 2014; Noda, 2014c; Seo et al., 2014). The most popularly applied external perturbation in 2DCOS is temperature (Kim et al., 2006b; Unger et al., 2009, 2011; Jia et al., 2010; Peng et al., 2010; Popescu and Vasile, 2010; Tang et al., 2010; Zheng et al., 2010; Cheng et al., 2011; Wang and Wu, 2011; Pazderka and Kopecký Jr, 2012; Chai et al., 2013; Li et al., 2013; Wang et al., 2013, 2014; Hoshina et al., 2014; Hou et al., 2014; Seo et al., 2014). Applications of 2DCOS in investigations of intriguing properties of polymer system measured by different types of analytical probes has been substantially increased (Izawa et al., 2001; Hong et al., 2005; Kim et al., 2006b; Jiang and Wu, 2008; Huang et al., 2009; Oh et al., 2009; Unger et al., 2009, 2011; Del Río et al., 2010; Huang and Kuo, 2010; Jelèić et al., 2010; Jia et al., 2010; Lee et al., 2010, 2012; Peng et al., 2010; Popescu and Vasile, 2010, 2011; Radice et al., 2010; Tang et al., 2010; Zheng et al., 2010; Cheng et al., 2011; Guo et al., 2011; Jin et al., 2011; Kuo and Liu, 2011; Ma et al., 2011; Wang and Wu, 2011; Zhang et al., 2011; Ando et al., 2012; Hoshina et al., 2012, 2014; Qu et al., 2012; Su et al., 2012; Wu et al., 2012; Zhong et al., 2012; Chai et al., 2013; Grzeszczuk et al., 2013; Lai and Wu, 2013; Park et al., 2013; Shinzawa et al., 2013; Wang et al., 2013, 2014; Hou et al., 2014; Noda, 2014c,d; Seo et al., 2014). Generalized 2D correlation spectra has notable advantages: examination of inter- or intra-molecular interactions and determination of the sequential order of events, which is hardly depicted in conventional spectroscopy.

In this review, the background of the generalized 2DCOS is briefly discussed, and the powerful applications of 2DCOS in the studies of polymers are presented. Illustrative examples of 2DOCS in polymer research describing the improved information gained with noteworthy developments of 2DCOS are also provided.

## Background

### Generalized 2D correlation spectroscopy

The detailed background of the generalized 2DCOS is well introduced in books and book chapters (Noda, 2002, 2009; Ozaki, 2002; Noda and Ozaki, 2004; Ozaki and Šašic, 2005; Ozaki and Noda, 2006; Noda and Lindsey, 2010; Czarnik-Matusewicz and Jung, 2014; Jung and Noda, 2014) and review articles (Noda et al., 1993, 2000; Noda, 2000, 2004, 2006, 2007, 2008, 2014a,b; Jung and Noda, 2014). Here, we briefly describe the basic concept of 2DCOS.

**Figure 1:**
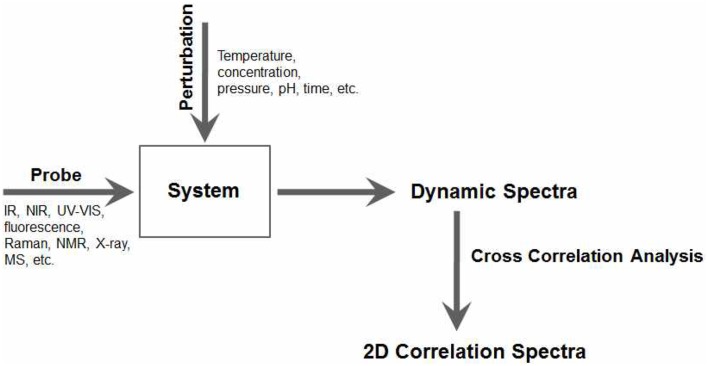
**The general scheme for constructing generalized 2D correlation spectra**.

In 2DCOS, a set of spectra *A*(ν_*j*_, *t*_*i*_) is obtained as a function of the spectral variable ν_*j*_ with **j** = 1, 2, … *n* and some perturbation variable *t_i_* with *i* = 1, 2, … *m* during a well-defined observation interval between *t*_1_ and *t_m_*. A series of perturbation-induced dynamic spectra collected in a systematic manner are transformed into a set of 2D correlation spectra by a simple cross correlation analysis as shown in Figure 1.

The *dynamic spectrum* Ã(ν*_j_*, *t_i_*) of a system induced by the application of an external perturbation is defined formally within the observation interval between *t*_1_ and *t_m_* as

(1) $$\tilde{A}\left(\nu_{j},t_{i}\right)\;=\;A\left(\nu_{j},t_{i}\right)-\bar{A}\left(v_{j}\right)$$

where *A*(*v_j_*) is the *reference spectrum* of the system. The reference spectrum is mostly selected as the *stationary* or *averaged spectrum* given by

(2) $$\bar{A}\left(\nu_{j}\right)\;=\;\frac{1}{m}\sum_{i=1}^{m}\;A\left(\nu_{j},t_{i}\right)$$

Synchronous Φ (ν_1_, ν_2_) and asynchronous Ψ (ν_1_, ν_2_) correlation spectra are given by

(3) $$\Phi (\nu_{1},\nu_{2})\;=\;\frac{1}{m-1}\sum_{i\;=\;1}^{m}\;\tilde{A}\left(\nu_{1},t_{i}\right)\cdot \tilde{A}\left(\nu_{2},t_{i}\right)$$

(4) $$\Psi (\nu_{1},\nu_{2})\;=\;\frac{1}{m-1}\sum_{i\;=\;1}^{m}\;\tilde{A}\left(\nu_{1},t_{i}\right)\cdot \sum_{k\;=\;1}^{m}N_{ik}\;\tilde{A}\left(\nu_{2},t_{k}\right)$$

where, *N_ik_* is the elements of so-called Hilbert–Noda transformation matrix given by

(5) $$N_{ik}\;=\{\;\begin{array}{cc} 0 & if\;i=k\\ \frac{1}{\pi (k-i)} & otherwise \end{array}\;$$

### 2D correlation spectra

The simultaneous or coincidental changes of spectral intensities at ν_1_ and ν_2_ are represented in the synchronous 2D correlation spectrum. Positive correlation peaks on the diagonal in synchronous 2D correlation spectrum correspond to the autocorrelation functions of spectral intensity variations, which are called autopeaks. Cross peaks, which are located at off-diagonal in synchronous 2D correlation spectrum, represent the coincidental or simultaneous changes of spectral intensities observed at two different spectral variables. The positive cross peaks depict that the intensities at corresponding spectral variables increase or decrease together. On the other hand, the negative cross peaks depict that one of the spectral intensities is increasing while the other is decreasing.

In contrast, the sequential, or unsynchronized, changes of spectral intensities at ν_1_ and ν_2_ are represented in the asynchronous 2D correlation spectrum. Asynchronous 2D correlation spectrum consists of only cross peaks, which shows an antisymmetric cross peaks with respect to the main diagonal. From the sign of cross peaks in 2D correlation spectra, the sequential changes in spectral intensities observed under the external perturbation can be determined. The same signs of synchronous and asynchronous cross peaks represent that the intensity change at ν_1_ occurs before ν_2_. While the different signs of synchronous and asynchronous cross peaks represent that the intensity change at ν_2_ occurs before ν_1_. This rule to determine sequential order of intensity variations is named Noda's rule.

## Application of 2DCOS in polymer studies

2DCOS, which can provide the easier access to the pertinent information in characterizing polymers, has been broadly applied to polymer studies to obtain new insights at the molecular level into the understanding behavior of polymers under an influence of an external perturbation. Various polymer systems, such as block copolymers (Kim et al., 2006b; Jia et al., 2010; Jin et al., 2011), biodegradable polymers (Guo et al., 2011; Unger et al., 2011; Ando et al., 2012; Hoshina et al., 2012, 2014; Wang et al., 2014), conducting polymer (Hong et al., 2005; Grzeszczuk et al., 2013), liquid crystals (Tang et al., 2010; Cheng et al., 2011), polymer blends (Oh et al., 2009; Unger et al., 2009, 2011; Jelèić et al., 2010; Popescu and Vasile, 2010, 2011; Kuo and Liu, 2011), and polymer nanocomposites (Huang et al., 2009; Huang and Kuo, 2010; Peng et al., 2010; Ando et al., 2012; Qu et al., 2012), etc., are analyzed by 2DCOS. Detailed information of polymers for polymerization (Izawa et al., 2001; Hong et al., 2005; Huang and Kuo, 2010; Qu et al., 2012; Noda, 2014d; Seo et al., 2014), melting behavior (Unger et al., 2009; Peng et al., 2010; Popescu and Vasile, 2010), crystallization (Huang et al., 2009; Zheng et al., 2010; Guo et al., 2011; Unger et al., 2011; Ando et al., 2012; Hoshina et al., 2012, 2014; Chai et al., 2013; Wang et al., 2013, 2014), gelation (Wang and Wu, 2011; Su et al., 2012; Park et al., 2013), photoreaction (Lee et al., 2010, 2012), hydration (Lai and Wu, 2013), sorption/desorption processes (Musto et al., 2011; Lai and Wu, 2013; Galizia et al., 2014), and phase transition/separation (Kim et al., 2006b; Cheng et al., 2011; Kuo and Liu, 2011; Hou et al., 2014), etc., which are undergoing under the influence of applied external perturbation, are obtained by 2DCOS. 2DCOS probes with various analytical techniques, such as IR, near-IR (NIR), Raman, X-ray, UV-Vis, THz, NMR spectroscopies, and even chromatography, have been successfully applied in polymer studies. 2DCOS has been extensively used in the IR study of polymers (Kim et al., 2006b; Huang et al., 2009; Unger et al., 2009, 2011; Del Río et al., 2010; Huang and Kuo, 2010; Jelèić et al., 2010; Jia et al., 2010; Lee et al., 2010, 2012; Peng et al., 2010; Popescu and Vasile, 2010, 2011; Zheng et al., 2010; Cheng et al., 2011; Jin et al., 2011; Kuo and Liu, 2011; Musto et al., 2011; Wang and Wu, 2011; Zhang et al., 2011; Ando et al., 2012; Qu et al., 2012; Su et al., 2012; Wu et al., 2012; Chai et al., 2013; Lai and Wu, 2013; Park et al., 2013; Shinzawa et al., 2013; Wang et al., 2013, 2014; Galizia et al., 2014; Hou et al., 2014; Noda, 2014c; Seo et al., 2014). Among the applications of 2DCOS in polymer study, temperature is the most used as an applied external perturbation (Kim et al., 2006b; Unger et al., 2009, 2011; Jia et al., 2010; Peng et al., 2010; Popescu and Vasile, 2010; Tang et al., 2010; Zheng et al., 2010; Cheng et al., 2011; Wang and Wu, 2011; Su et al., 2012; Wang et al., 2013, 2014; Hoshina et al., 2014; Hou et al., 2014; Seo et al., 2014).

Here, several illustrative examples are presented to demonstrate the utility of 2DCOS in polymer studies. Special techniques in 2DCOS, such as hetero-spectral correlation, positive null-space projection, and 2DOCS combined with chemometric methods are discussed.

### 2D IR correlation spectroscopy

In the field of polymer study through 2DCOS, IR spectroscopy is the most commonly used analytical probes. The advantages of 2DCOS, such as the enhanced spectral resolution and determination of sequential changes of spectral band intensities, can provide the useful information in characterizing structural changes of polymer obtained as a function of a perturbation, which is not readily observed in the conventional spectroscopy (Kim et al., 2006b; Huang et al., 2009; Unger et al., 2009, 2011; Del Río et al., 2010; Huang and Kuo, 2010; Jelèić et al., 2010; Jia et al., 2010; Lee et al., 2010, 2012; Peng et al., 2010; Popescu and Vasile, 2010, 2011; Zheng et al., 2010; Cheng et al., 2011; Jin et al., 2011; Kuo and Liu, 2011; Wang and Wu, 2011; Zhang et al., 2011; Ando et al., 2012; Qu et al., 2012; Su et al., 2012; Wu et al., 2012; Chai et al., 2013; Lai and Wu, 2013; Park et al., 2013; Shinzawa et al., 2013; Wang et al., 2013, 2014; Hou et al., 2014; Noda, 2014c; Seo et al., 2014).

Popular perturbations in polymer studies are temperature (Kim et al., 2006b; Unger et al., 2009, 2011; Jia et al., 2010; Peng et al., 2010; Popescu and Vasile, 2010; Tang et al., 2010; Zheng et al., 2010; Cheng et al., 2011; Wang and Wu, 2011; Su et al., 2012; Wang et al., 2013, 2014; Hoshina et al., 2014; Hou et al., 2014; Seo et al., 2014), concentration (Huang et al., 2009; Jelèić et al., 2010; Kuo and Liu, 2011; Popescu and Vasile, 2011; Wu et al., 2012; Grzeszczuk et al., 2013), time (Huang and Kuo, 2010; Jin et al., 2011; Ando et al., 2012; Lee et al., 2012; Qu et al., 2012; Su et al., 2012; Lai and Wu, 2013; Park et al., 2013; Noda, 2014c), and pressure (Zhang et al., 2011; Shinzawa et al., 2013), etc. Especially, temperature is the most commonly used because ordinary changes in density, which give rise to nonspecific spectral changes, usually are accompanied by structural changes in polymer upon heating or cooling.

**Figure 2:**
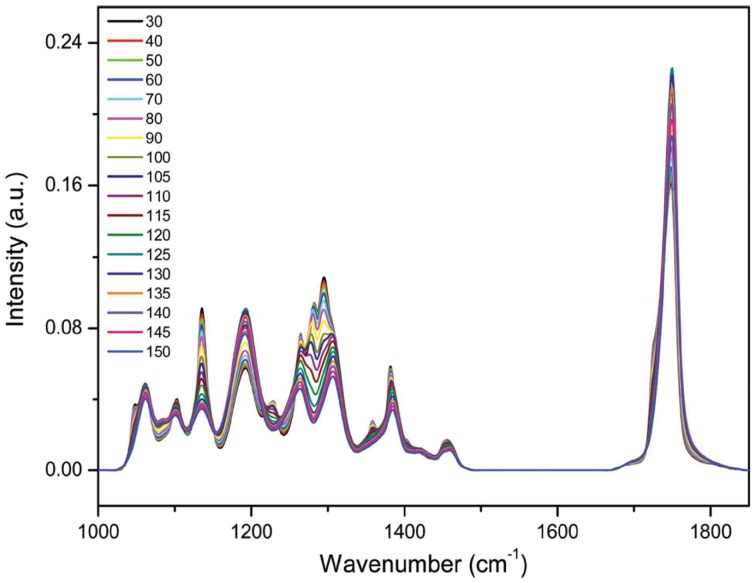
**IRRAS spectra of a spin-coated film of P(HB-*co*-HHx) (HHx = 12.0 mol%) during heating from 30 to 150°C at an interval of 5°C.** (Reproduced with permission *J. Phys. Chem. B* 2010, 14, 10979–10985, Copyright 2010, American Chemical Society).

**Figure 3:**
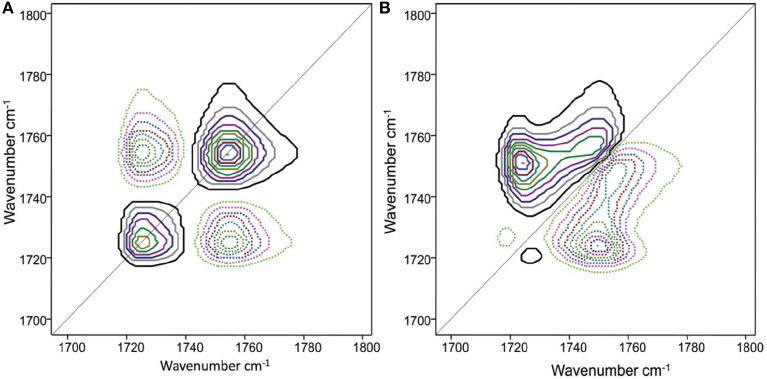
**Synchronous (A) and asynchronous (B) 2D correlation spectra obtained from the temperature-dependent IRRAS spectra of a spin-coated film of P(HB-*co*-HHx) (HHx = 12.0 mol%)**. The solid and dashed lines represent positive and negative cross peaks, respectively. (Reproduced with permission *J. Phys. Chem. B* 2010, 14, 10979–10985, Copyright 2010, American Chemical Society).

Choi et al. demonstrated the details of thermal behavior of spin-coated films of biodegradable poly(3-hydroxybutyrate-*co*-3-hydroxyhexanoate) or P(HB-*co*-HHx) (HHx = 12.0, 10.0, 3.8 mol%) copolymers by using 2DCOS (Choi et al., 2010). The temperature-dependent infrared-reflection absorption (IRRAS) spectra of a spin-coated film of P(HB-*co*-HHx) (HHx = 12.0 mol%) copolymer, which were measured during the heating process, are shown in Figure 2. Two distinct C=O stretching bands, a crystalline band and an amorphous band are observed respectively at 1726 cm^−1^ and near 1751 cm^−1^. The 2D correlation spectra for the C=O stretching bands are shown in Figure 3. Two main bands are observed at 1726 and 1751 cm^−1^ assigned to the crystalline band and the amorphous band, respectively, in the synchronous 2D correlation spectrum. Interestingly the crystalline band at 1726 cm^−1^ observed in synchronous 2D correlation spectrum is clearly resolved into two bands at 1721 and 1730 cm^−1^ in the asynchronous 2D correlation spectrum, which is hardly detectable in the original IRRAS spectra shown in Figure 2. A band observed at a lower wavenumber corresponds to the well-ordered primary crystals and the other at a higher wavenumber corresponds to less ordered secondary crystals. From the analysis of the sign of cross peaks in 2D correlation spectra, they determined the sequential order of spectral changes with increasing temperature that the intensity of an amorphous band changes first and then that for less ordered secondary crystals changes before that for well-ordered secondary crystals.

The application of 2DCOS in the analysis of transport phenomena in polymers (Musto et al., 2011; Lai and Wu, 2013; Galizia et al., 2014) can provide useful insights about the distribution of penetrant in polymer matrix, which is of great practical relevance in several applications, such as membrane science, drug delivery, and polymer durability. Musto et al. applied 2D IR correlation spectroscopy for the investigation of the diffusion process and the sorption equilibrium of water vapor in polyimide films (Musto et al., 2011). The molecular level characterization of the mass-transport process and the sorption thermodynamics were detected. They also investigated the diffusion mechanism in biocompatible thermoplastic polymer, poly-e-caprolactone (PCL), by using 2D IR correlation spectroscopy, and the sorption-desorption cycle for a molecular level characterization of the H_2_O/PCL system and molecular interaction formed (H-bonding) were detected (Galizia et al., 2014). Lai and Wu reported the water sorption and desorption processes with a pharmaceutical amphiphilic copolymer poly(3-(2-methoxyethyl)-N-vinyl-2-pyrrolidone) investigated by using 2D IR correlation spectroscopy (Lai and Wu, 2013). Different states of hydrogen-bonded water molecules were also detected in 2D IR correlation spectra.

**Figure 4:**
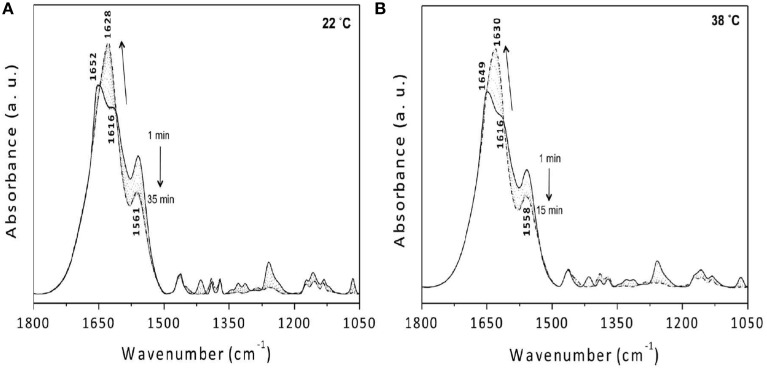
**ATR-FTIR spectra of the NiPAAm gelation process measured at 22 (A) and 38°C (B)**. Spectra are collected at every 1 min. The solid and dashed lines represent, respectively, the spectrum measured at 1 and 35 min **(A)** and 1 and 15 min **(B)** after initiation of the gelation reaction. The dotted lines represent the time evolution of the spectra in between the first and last spectra, and the arrows indicate the trend for the band intensity change with the upward arrow and the downward arrow showing the intensity increase and decrease with time, respectively. (Reproduced with permission *Macromolecules* 2013, 46, 3587–3602, Copyright 2013, American Chemical Society).

**Figure 5:**
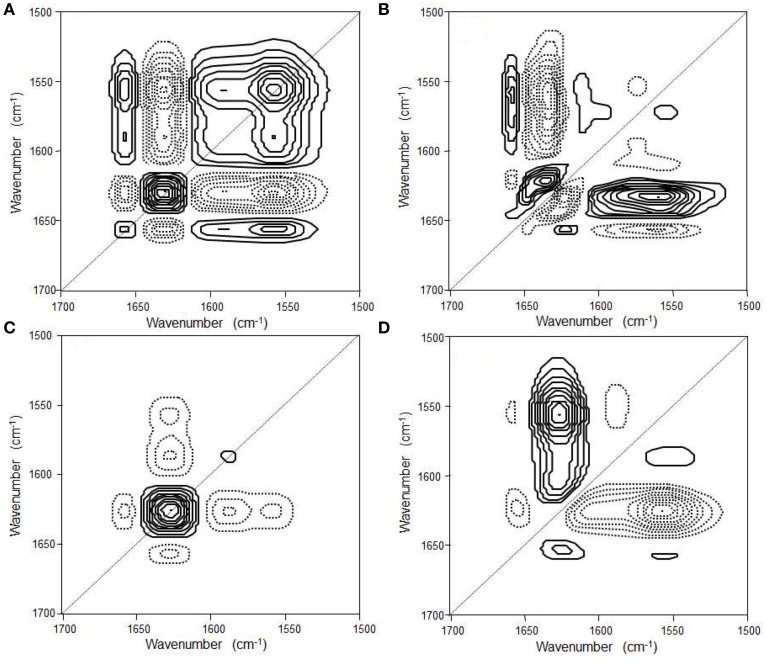
**Synchronous (A) and asynchronous (B) 2D correlation spectra in the first-stage reaction process and synchronous (C) and asynchronous (D) 2D correlation spectra in the second-stage reaction process of the NiPAAm gelation process at 22°C**. The solid and dashed lines represent positive and negative cross peaks, respectively. (Reproduced with permission *Macromolecules* 2013, 46, 3587–3602, Copyright 2013, American Chemical Society).

**Figure 6:**
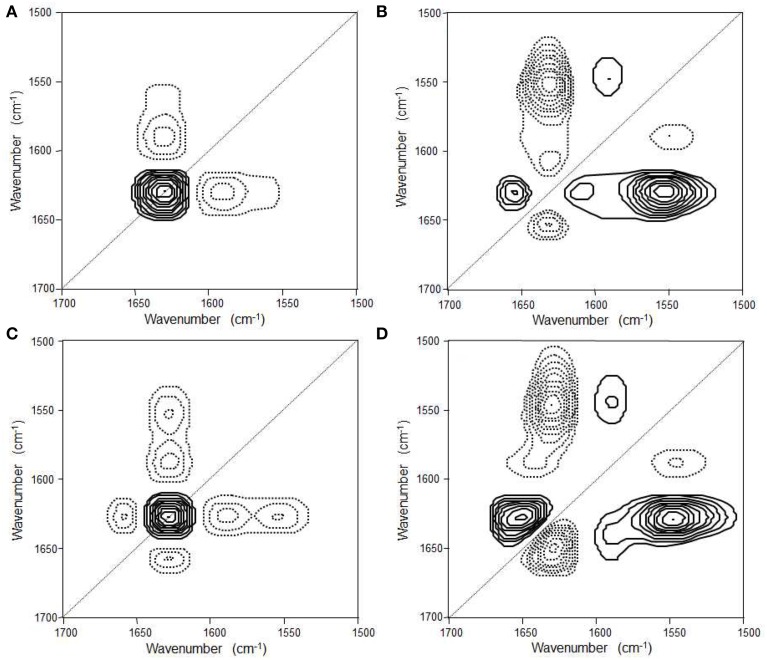
**Synchronous (A) and asynchronous (B) 2D correlation spectra in the first-stage reaction process and synchronous (C) and asynchronous (D) 2D correlation spectra in the second-stage reaction process of the NiPAAm gelation process at 38°C**. The solid and dashed lines represent positive and negative cross peaks, respectively. (Reproduced with permission *Macromolecules* 2013, 46, 3587–3602, Copyright 2013, American Chemical Society).

Park et al. reported the mechanism of chemical gelation process of poly(*N*-isopropylacrylamide) (PNiPAAm) hydrogel by using *in situ* observations with time-resolved FTIR and 2DCOS at two characteristic preparation temperatures below and above the lower critical solution temperature (LCST) of PNiPAAm aqueous solution (Park et al., 2013). Figures 4A,B show the FTIR spectra in the 1800-1050 cm^−1^ region for the NiPAAm gelation process measured at *T_p_* = 22 and 38°C, respectively. The spectral changes during the NiPAAm gelation process at the two different temperatures were qualitatively similar except for the differences associated with the time required for completing the gelation reaction (~30 and ~15 min at *T_p_* = 22 and 38°C, respectively). The bands at 1628 (Figure 4A) and 1630 cm^−1^ (Figure 4B) started to appear at ~20 and ~8 min after the onset of the reaction, respectively. Those bands became increasingly remarkable with time and most prominent at the end of the gelation process, independent of *T_p_s*. It identifies the specific time-spans for two stage reaction process: the first-stage giving rise to linear and branched random copolymers of NiPAAm and cross-linker monomers and the second-stage giving rise to cross-linking into macroscopic network structure. 2DCOS was thus applied both the first-stage and second-stage reaction processes to better elucidate the gelation process. The each stage of 2D correlation spectra for the NiPAAm gelation process at 22 and 38°C, respectively, shown in Figures 5, 6 are completely different, although IR spectra obtained below and above LCST are apparently similar. From the analysis of 2D correlation spectra, they firstly identified the specific time span for each stage of the two-stage reaction process at two temperatures. Two different gelation process below and above LCST are summarized in Tables 1, 2.

**Table 1:**
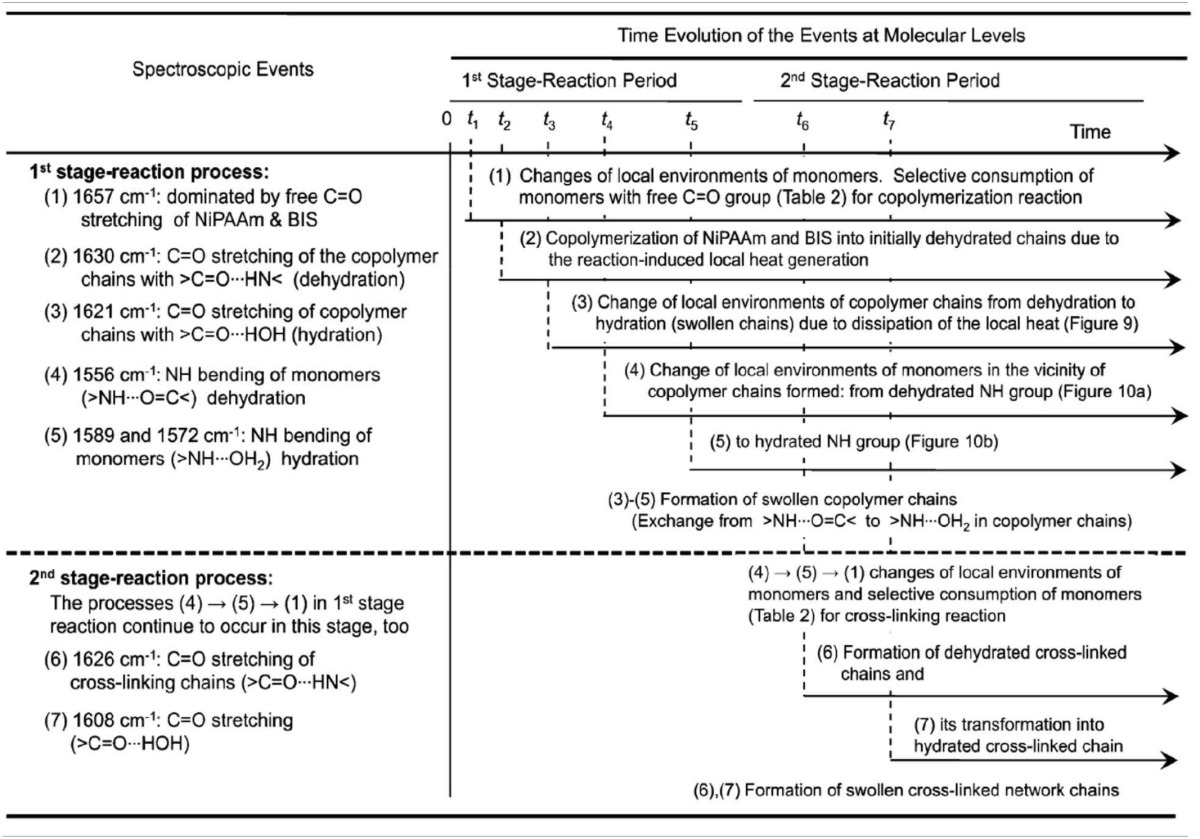
**Molecular significance of sequence changes of the spectroscopic events with time for two-stage chemical gelation processes below LCST (*T_p_* = 22°C) as observed by the 2D IR correlation spectroscopy**.

*Reproduced with permission Macromolecules 2013, 46, 3587-3602, Copyright 2013, American Chemical Society*.

**Table 2:**
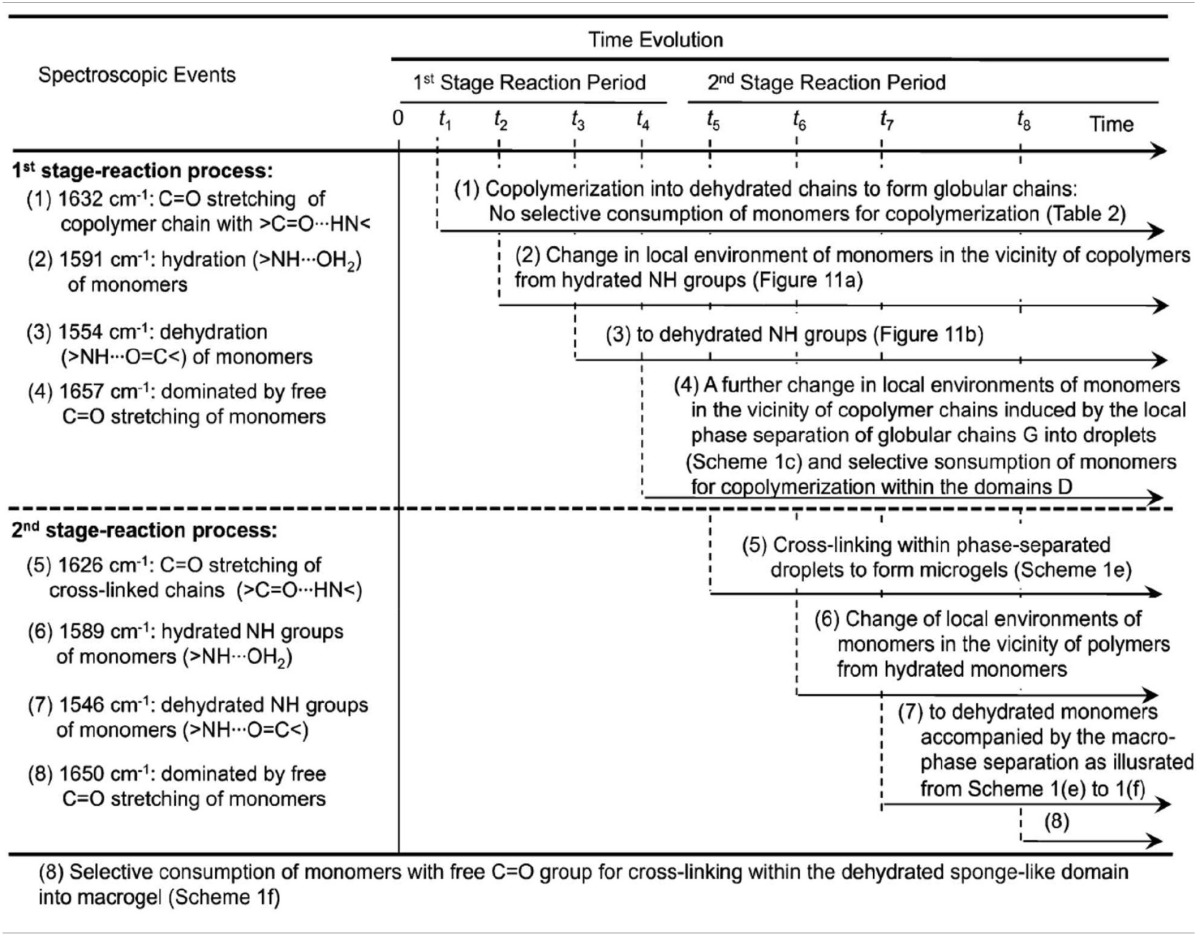
**Molecular significance of sequence changes of the spectroscopic events with time for two-stage chemical gelation processes below LCST (*T_p_* = 38°C) as observed by the 2D IR correlation spectroscopy**.

*Reproduced with permission Macromolecules 2013, 46, 3587-3602, Copyright 2013, American Chemical Society*.

### 2D hetero-spectral correlation analysis

The hetero-correlation analysis provides a very powerful advantage to 2DCOS. Three possible possibilities in 2D hetero-correlation analysis are hetero-spectral correlation (Kim et al., 2006a; Choi et al., 2010; Katayama et al., 2010; Smirnova et al., 2011; Park et al., 2012; Ryu et al., 2012; Shinzawa et al., 2012), hetero-perturbation (or hybrid) correlation (Wu et al., 2002, 2006), and hetero-sample correlation (Czarnik-Matusewicz et al., 2009; Pi et al., 2010). Among them, 2D hetero-spectral correlation is the most active field in applications of 2D hetero-correlation analysis. It can compare two completely different types of spectral data obtained for a system under a similar external perturbation. In the 2D hetero-spectral correlation analysis, the correlation between different spectral signals under the same perturbation can be detected. It is possible to apply 2D hetero-spectral analysis to the correlation not only between closely related spectroscopic measurement, such as IR and Raman spectra, but also between completely different types of spectroscopic or physical techniques, such as IR and X-ray spectroscopy.

**Figure 7:**
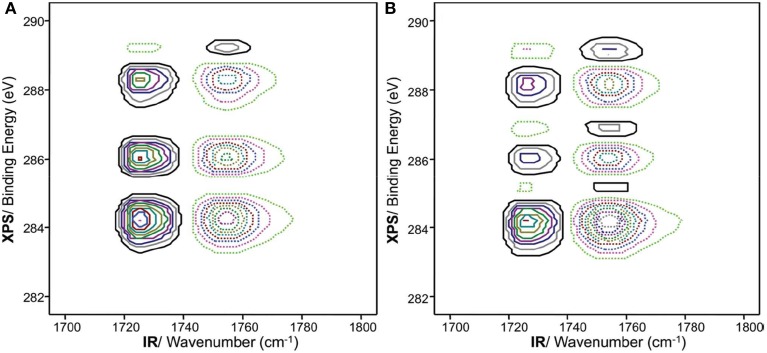
**Synchronous (A) and asynchronous (B) hetero-spectral 2D XPS/IR correlation spectrum for a spin-coated film of P(HB-*co*-HHx) (HHx = 12.0 mol%)**. The solid and dashed lines represent positive and negative cross peaks, respectively. (Reproduced with permission *J. Phys. Chem. B* 2010, 14, 10979–10985, Copyright 2010, American Chemical Society).

Choi et al. used 2D hetero-spectral correlation between completely different types of spectroscopy, such as IR and X-ray photoelectron spectroscopy (XPS), for the investigation of thermal behavior of biodegradable copolymers under increasing temperature (Choi et al., 2010). The 2D hetero-spectral IR/XPS correlation spectra of spin-coated film of P(HB-*co*-HHx) copolymer during heating process are shown in Figure 7. In synchronous 2D hetero-spectral IR/XPS correlation spectrum, two XPS band at 289.3 eV and near 288.3 eV, which are assigned to amorphous and crystalline components, respectively, are clearly observed. Very interestingly, asynchronous 2D hetero-spectral IR/XPS correlation spectrum reveals the sequential order of the intensity changes that spectral intensity changes detected by the IR probe always occurred earlier than those by XPS. This result provides that the thermal phase transition of P(HB-*co*-HHx) copolymer actually involves different level of microscopic scales. That is because IR probe detects long range molecular interactions while XPS detects more localized structure changes during the gradual melting process. This probe-dependent asynchronicity, which is spectral changes of IR probe appear first before those of XPS, clearly reflects the subtle difference in the selectivity and specificity of these probes toward molecular scale changes under the same external perturbation. The 2D hetero-spectral IR/XPS correlation analysis sheds light on the correlation between IR and XPS spectral changes, which is difficult to detect from a simple analysis of IR or XPS spectra alone.

### Projection 2D correlation analysis

Noda has proposed a new generation technique of 2DCOS (Noda, 2010). Projection 2D correlation analysis can dramatically simplify highly congested 2D correlation spectra often encountered. This technique is based on the use of mathematical matrix projection to selectively filter out the unwanted portion of the information of spectral data. The combination of the projection and null-space projection operations might be a very useful technique to augment or attenuate select features within congested 2D correlation spectra for easier interpretation. Details of background of projection 2D analysis was previously described (Noda, 2010).

Here we will briefly provide the basic concept of projection 2D correlation spectra which is based on terms of a series of matrix manipulations (Noda, 2010). In generalized 2DCOS, spectral data matrix **A** (*m* × *m*) consisting of *m* rows of spectra with *n* columns of spectral variables, like wavenumber, represents the system response. For convenience, each column of the matrix **A** corresponds to dynamic spectrum Ã(ν_*j*_, *t_i_*) used in Equations (3) and (4) scaled by square root of 1/(*m* – 1). Generalized 2D correlation spectra can be obtained by a simple matrix multiplication applied toward the spectral data matrix. The synchronous and asynchronous correlation spectra, **Φ** and Ψ, are then obtained as

(6) $$\Phi =\;{\text{A}}^{\text{T}}\text{A}$$

(7) $$\Psi =\;{\text{A}}^{\text{T}}\text{NA}$$

As already indicated in Equation (5), **N** is the so-called Hilbert–Noda transformation matrix.

In projection analysis, an arbitrary *m* × *m* matrix **Y**, which is different from the spectral data matrix **A**, define the projection matrix **R**_Y_ of **Y** as

(8) $${\text{R}}_{\text{Y}}=\text{Y}{\left({\text{Y}}^{\text{T}}\text{Y}\right)}^{-1}{\text{Y}}^{\text{T}}$$

The superscripts ^T^ and ^−1^, respectively, stand for the transpose and inverse operation of the matrix.

The spectral data matrix **A** can be transformed to a new form of data matrix by the projection operation. The projected data matrix **A**_P_ is obtained by the simple multiplication of **R**_Y_ with **A**,

(9) $${\text{A}}_{\text{P}}=\;{\text{R}}_{\text{Y}}\text{A}$$

The newly obtained projected data matrix **A**_P_ represents the matrix projection of **A** onto an abstract mathematical space spanned by the columns of **Y**. In other words, **A**_P_ is the closest possible reconstruction of **A** by using only the linear combinations of all the columns of **Y**. To make this operation possible, matrices **A** and **Y** must have the same number of rows *m*. It is actually common to select **Y** from several select columns of **A**.

The corresponding null-space projection is carried out as

(10) $${\text{A}}_{\text{N}}=\left(\text{I}-{\text{R}}_{\text{Y}}\right)\text{A}=\text{A}-{\text{A}}_{\text{P}}$$

The null-space projected data matrix **A**_N_ represents the projection of **A** onto the space spanned by vectors which are orthogonal to the columns of **Y**. In other words, **A**_N_ is the residual after the removal of **A**_P_ from **A**. Thus, the projection operations separate the original data into two orthogonal parts, **A** = **A**_P_ + **A**_N_, by using the information contained within the chosen matrix **Y**.

Data matrices created by various projection-based transformation operations discussed above can be readily converted to 2D correlation spectra. For example, by using Equation (9), it is possible to obtain the 2D correlation spectra for the projected data matrix **A**_P_

(11) $$\Phi_{\text{p}}=\;{\text{A}}_{\text{P}}{}^{\text{T}}{\text{A}}_{\text{P}}=\;{\text{A}}^{\text{T}}{\text{R}}_{\text{Y}}\text{A}$$

(12) $$\Psi_{\text{p}}=\;{\text{A}}_{\text{P}}{}^{\text{T}}{\text{NA}}_{\text{P}}=\;{\text{A}}^{\text{T}}{\text{R}}_{\text{Y}}{\text{NR}}_{\text{Y}}\text{A}$$

The term **R**_Y_ appears in Equation (11) only once because this matrix is idempotent. 2D correlation spectra **Φ**_p_ and Ψ_p_ for the projected data provide the correlation information among select signals of **A**, which are in turn correlated with the projector matrix **Y**. In other words, all other signals not correlated **Y** will be filtered out prior to the 2D correlation analysis.

Correlation analysis of the null-space projected data, **A**_N_ = **A** – **A**_P_, results in the following set of 2D spectra.

(13) $$\Phi_{\text{N}}=\;{\text{A}}_{\text{N}}{}^{\text{T}}{\text{A}}_{\text{N}}={\text{A}}^{\text{T}}\left(\text{I}-{\text{R}}_{\text{Y}}\right)\text{A}$$

(14) $$\Psi_{\text{N}}=\;{\text{A}}_{\text{N}}{}^{\text{T}}{\text{NA}}_{\text{N}}={\text{A}}^{\text{T}}\left(\text{I}-{\text{R}}_{\text{Y}}{\text{NR}}_{\text{Y}}-{\text{NR}}_{\text{Y}}-{\text{R}}_{\text{Y}}\text{N}\right)\text{A}$$

**Figure 8:**
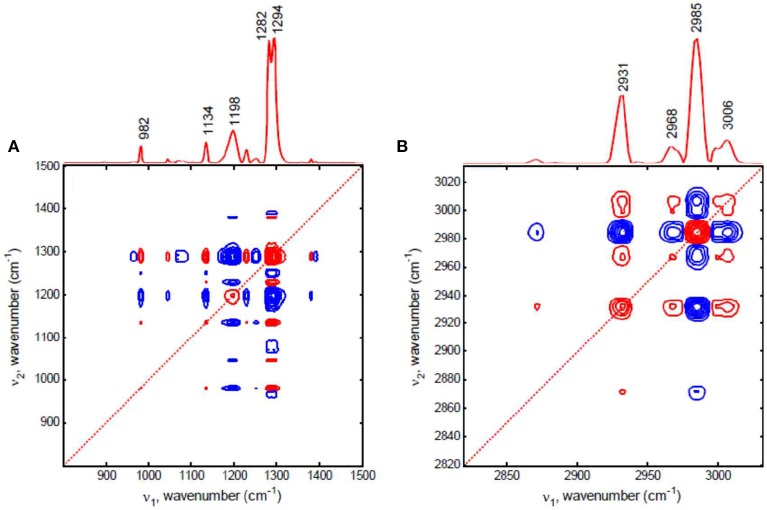
**Synchronous conventional 2D correlation spectra in the region of C-H deformation, C-O-C stretching, and C=O stretching modes in 1000–1500 cm^−1^ (A) and C-H stretching mode in 2800–3050 cm^−1^ (B) for spin-coated film of P(HB-*co*-HHx)/PEG blend, respectively**. The red and blue lines represent positive and negative cross peaks, respectively. (Reproduced with permission *Vib. Spectrosc*. 2012, 60, 163–167, Copyright 2012, Elsevier).

**Figure 9:**
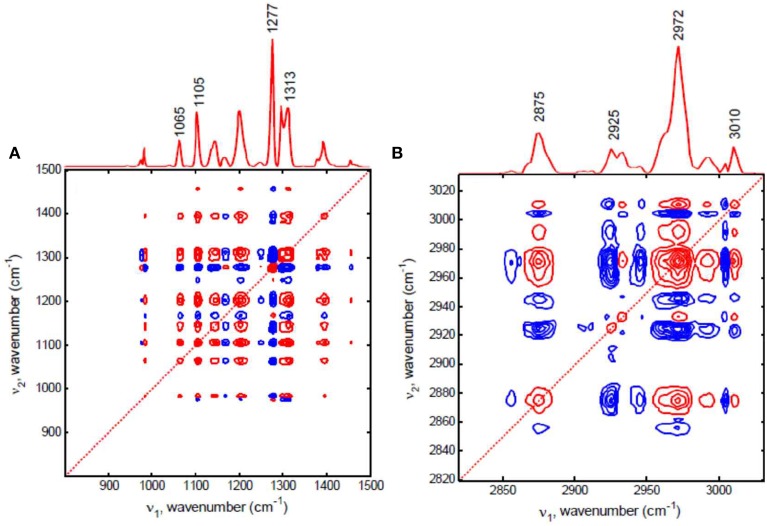
**Synchronous null-space projection 2D correlation spectra in the region of C-H deformation, C-O-C stretching, and C=O stretching modes in 1000–1500 cm^−1^ (A) and C-H stretching mode in 2800–3050 cm^−1^ (B) for spin-coated film of P(HB-co-HHx)/PEG blend, respectively**. The red and blue lines represent positive and negative cross peaks, respectively. (Reproduced with permission *Vib. Spectrosc*. 2012, 60, 163–167, Copyright 2012, Elsevier).

Kim et al. (2012) reported the dominant crystalline contribution in biodegradable polymer blend with temperature increase was successfully filtered out by using the null-space projection, which can extract other finer details. Figure 8 shows the conventional synchronous 2D correlation spectra of spin-coated film of poly(3-hydroxybutyrate-*co*-3-hydroxyhexanoate)/polyethylene glycol (P(HB-*co*-HHx)/PEG) blend. In conventional 2D correlation spectra, all spectral changes are contributed from not PEG but P(HB-*co*-HHx) in P(HB-*co*-HHx)/PEG blend during heating process. They performed null-space projection 2D correlation analysis to selectively filter out the contribution of P(HB-*co*-HHx). As shown in Figure 9, the synchronous null-space projection 2D correlation spectra, which are constructed from the null-space projected data with the crystalline signals of P(HB-*co*-HHx) removed, are completely different with conventional 2D correlation spectra. The observed new bands at 1313, 1105, and 1065 cm^−1^ in Figure 9A, which are hardly detected in conventional 2D correlation spectrum, can be assigned to PEG. In Figure 9B, two bands at 2972 and 2875 cm^−1^, which are not observed in conventional 2D correlation spectrum, can also be assigned to PEG. The subtle contribution of PEG in spin-coated film of P(HB-*co*-HHx)/PEG blend during heating process is clearly detected in null-space 2D projection correlation spectra.

### Combination of 2DCOS and chemometric techniques

The fruitful combination of 2DCOS and chemometric techniques often provides more useful information to interpret subtle spectral changes of system, which is barely detected in conventional 2DCOS (Jung, 2004; Jung et al., 2002, 2003a,b,c,d, 2006). Jung *et al*. introduced the possible combination of 2DCOS and principal component analysis (PCA). In this technique named PCA 2DCOS, PCA is an essential and integral part of the subsequent 2DCOS (Jung, 2004; Jung et al., 2002). Jung et al. also reported a new concept of *engenvalue manipulating transformation* (EMT) for PCA 2DCOS (Jung et al., 2003b,c,d, 2006).

A brief background of PCA 2DCOS and EMT are provided here. The original set of perturbation-dependent spectral data matrix **A** is an *n* × *m* matrix with *n* spectra and *m* wavenumber points. In PCA, the significant part of the data matrix A^*^ can be expressed as the product of score and loading matricies

(15) $$A=WV^{T}+E=A^{*}+E$$

where **W** and **V** are the loading matrix (*m* × *r*) and score matrix (*n* × *r*), respectively, and **E** is the residual matrix often related with pure noise. The matrix product A^*^ is the noise-free reconstructed data matrix of the original data **A**.

(16) $$A^{*}=W\;V^{T}$$

In PCA 2DCOS, this *reconstructed data matrix* A^*^ is used instead of the original data matrix. PCA 2DCOS reconstructed from a few selected significant scores and loading vectors of PCA can accentuate only the most important features of synchronicity and asynchronicity without noise contribution. It is a very powerful technique for eliminating noise contribution from the spectra to extract useful information.

The PCA-reconstructed data matrix A^*^ can be also expressed in the form of singular value decomposition (SVD),

(17) $$A^{*}=US\;V^{T}$$

and

(18) $$S=L^{1/2}$$

where **U** and **S** are the orthonormal score matrix and diagonal matrix containing the singular value, respectively.

Here **L** = **W′ W** is a diagonal matrix where each diagonal element corresponds to the eigenvalue of principal component. The score matrix **W** is expressed in the form **W** = **U S** and can be obtained directly from **W** = **A V**.

By manipulating and replacing eigenvalues of A^*^, the new transformed data matrix A^**^ can be obtained

(19) $$A^{**}=US^{**}\;V^{T}$$

where S^**^ is given by varying the corresponding eigenvalues in **S** by raising them to the power of m.

(20) $$S^{**}=S^{m}$$

The new *EMT-reconstructed data matrix* A^**^ is used instead of A^*^ to enhance 2D correlation spectra. The smaller eigenvalues becomes more prominent, by uniformly lowering the power of a set of eigenvalues associated with the original data. In this technique, the contributions of minor components but potentially important factors is amplified.

**Figure 10:**
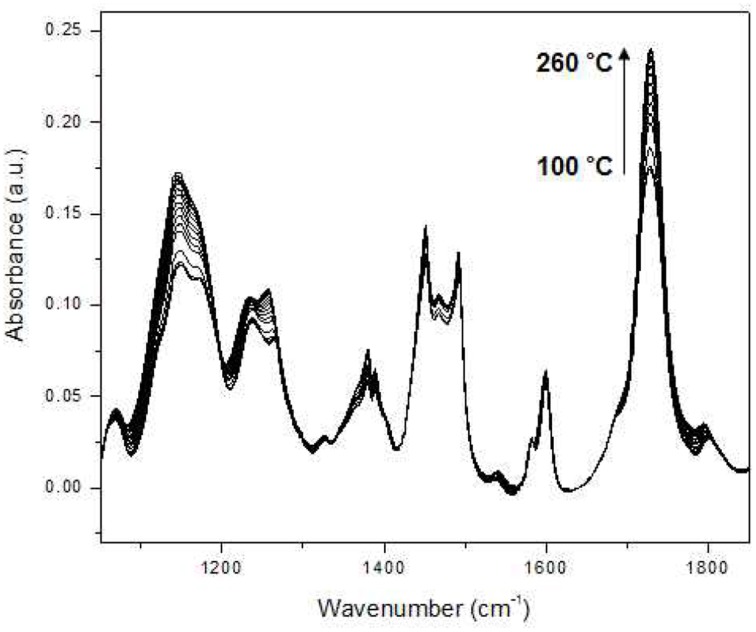
**Temperature-dependent IR spectra of polystyrene-*block*-poly(*n*-pentyl methacrylate) (PS-PnPMA) measured during heating from 100 to 260°C at an interval of 5°C**. (Reproduced with permission *J. Mol. Struct*. 2006, 799, 96–101, Copyright 2006, with permission from Elsevier).

**Figure 11:**
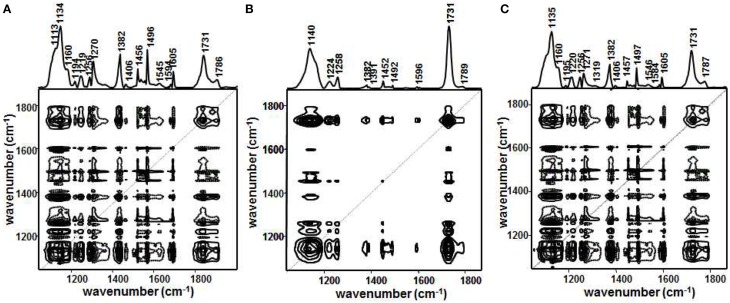
**Conventional synchronous 2D correlation spectra obtained from the raw spectra in Figure 10 for disordered state in lower temperature (A), ordered state (B), and disordered state in higher temperature (C), respectively**. Solid and dashed lines represent positive and negative cross peaks, respectively. (Reproduced with permission *Macromolecules* 2006, 39, 408–412, Copyright 2006, American Chemical Society).

**Figure 12:**
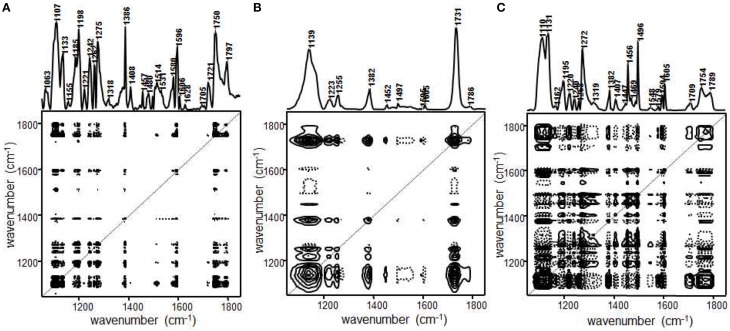
**Synchronous PCA 2D correlation spectra obtained from the EMT reconstructed data with *m* = 1/2 for disordered state in lower temperature (A), ordered state (B), and disordered state in higher temperature (C), respectively**. Solid and dashed lines represent positive and negative cross peaks, respectively. (Reproduced with permission *J. Mol. Struct*. 2006, 799, 96–101, Copyright 2006, with permission from Elsevier).

Jung et al. demonstrated that PCA 2DCOS through EMT technique was performed to more clearly understand the phase behavior of polystyrene-*block*-poly(*n*-pentyl methacrylate) (PS-PnPMA) (Jung et al., 2006). PS-PnPMA is a very interesting closed-loop block copolymer, which has a lower disorder-to-order transition (LDOT) temperature and an upper order-to-disorder transition (UODT) temperature. The temperature-dependent IR spectra of PS-PnPMA measured during heating from 100–260°C are shown in Figure 10. In the conventional 2D IR correlation spectra shown in Figure 11, the ordered state is completely different with two disordered states and these two disordered states at lower and higher temperatures are also different (Kim et al., 2006b). To highlight subtle differences of the two disordered states of PS-PnPMA, they applied PCA 2DCOS through EMT method to the temperature-dependent IR spectra. In PCA analysis, the original spectral data set shown in Figure 10 was decomposed into the scores and loading vectors. Synchronous PCA 2D correlation spectra generated from the reconstructed data matrix A^*^ with the three principal components are like the conventional 2D correlation spectra but without noise contribution. Figures 12A–C shows synchronous PCA 2D correlation spectra generated from the EMT-reconstructed spectral data matrix A^**^ obtained by replacing the original eigenvalues with *m* = 1/2 for disordered state at lower temperature, ordered state, and disordered state at higher temperature, respectively. By lowering the power of a set of eigenvalues associated with the original data, hidden property of phase transition from the contribution of minor but potentially interesting is much more greatly accentuated than conventional 2D correlation spectra. As shown in Figure 12, synchronous spectrum generated from the EMT-reconstructed spectral data matrix of the ordered state is completely different from those in the two disordered states and the clear difference between two disordered states is also observed. In the power spectrum, extracted along the diagonal line of the synchronous 2D correlation spectrum, in the top of Figures 12A,C, intensities of bands from C-C-O stretching, C-H deformation, and C=O stretching of PnPMA change greatly at lower temperature while those from phenyl group in PS change greatly at higher temperature. The distinct difference in two disordered states in the cross correlations of the bands from phenyl group in PS with that from C-C-O group in PnPMA reveals that the conformation of PS-PnPMA and the weak interaction between phenyl group of PS and the side chain of PnPMA in the two disordered states are different. The EMT technique clearly distinguish the very subtle differences of spectra which are not observed in conventional 2D correlation spectra.

Jung et al. also demonstrated the use of 2DCOS in conjunction with alternating least squares (ALS) based self-modeling curve resolution (SMCR) analysis of spectral data sets (Jung et al., 2003a). In this iterative regression technique, asynchronous 2D correlation peaks for the identification of pure variables were used as the initial estimates in the ALS process. Choosing the most distinct bands via the positions of asynchronous 2D peaks is a viable starting point for ALS iteration (Jung et al., 2003a; Hong et al., 2005). Once the pure variables are selected, ALS regression can be used to obtain the concentration profiles and pure component spectra.

Hong et al. studied the electrochemical polymerization of aniline by using real-time spectroelectrochemical experiments conducted concurrently with potentiodynamic scans (Hong et al., 2005). They performed 2DCOS and subsequent extraction of pure component spectra as well as their relative concentration profiles from complex spectroelectrochemical data employing the ALS-based SMCR method. All of the spectra of intermediate species proposed in the literature are identified. The concentrations of intermediate species varying as a function of the scanned potential are also determined. It was the first complete analysis of the complex spectra of aniline oxidation, which provides full understanding of the aniline polymerization reaction.

## Summary

2DCOS has become a very popular tool in the field of polymer study. It can be utilized with a number of spectroscopic and other analytical probes for a very broad range of polymer systems by employing different types of external perturbations to induce spectral variations. This review covers the basic concept of generalized 2DCOS and noteworthy progress in 2DCOS and their applications in polymer study. New developments in 2DCOS provide a powerful analytical technique applicable to the in-depth analysis of various spectral data. Active and steady progress in 2DCOS would open a way for studying polymers in many applications.

### Conflict of interest statement

The authors declare that the research was conducted in the absence of any commercial or financial relationships that could be construed as a potential conflict of interest.
